# USING LONGITUDINAL HEALTH INFORMATION TO ENHANCE MORTALITY PREDICTION

**DOI:** 10.1093/geroni/igz038.023

**Published:** 2019-11-08

**Authors:** Megan K Beckett, Marc N Elliott, Doug Ritenour, Laura A Giordano, Debra Saliba

**Affiliations:** 1 RAND Corporation, Santa Monica, California, United States; 2 Health Services Advisory Group, Phoenix, Arizona, United States

## Abstract

The Vulnerable Elders Survey-13-Health Outcomes Survey (VES-13-HOS) 2.5 is a simple, validated method to predict two-year mortality using older adults’ responses to the Medicare HOS. We explore whether adding longitudinal function measures and expanded sociodemographic information significantly improves the performance of the VES-13-HOS 2.5. Using Medicare HOS data, we developed risk scores that used coefficients from three logistic regressions predicting two-year mortality (2013-2015): (1) standard 0-10 VES-HOS 2.5 inputs at 2013 (function and limited demographics), (2) expanded demographics at 2013 (adding gender, fine-grained age, race/ethnicity, marital status, missing income indicator, and Medicaid status), (3) expanded demographics at 2013 plus change in pre-baseline (2011) health score two years prior. Fine-grained age and sociodemographic information slightly improve VES mortality prediction. Holding the 2013 VES-HOS 2.5 0-18 enhanced-demographic risk score constant, seniors with worse 2011 than 2013 functioning had higher two-year mortality than seniors with better 2011 functioning. Adding this prior health status further improved model performance slightly- 80% of mortality prediction is explained by “current” health and function and 20% by status two years earlier; such weighted scoring could be employed. We find that prior health status measurements do not generally indicate a trajectory that is likely to continue. Rather, health and function information from two years prior reduces measurement error via a second assessment of health; those with much worse health and function two years earlier are at slightly higher mortality risk. The VES-HOS can be used to identify patients with high mortality risk and to guide their care.

